# *Marmota himalayana* in the Qinghai–Tibetan plateau as a special host for bi-segmented and unsegmented picobirnaviruses

**DOI:** 10.1038/s41426-018-0020-6

**Published:** 2018-03-07

**Authors:** Xue-lian Luo, Shan Lu, Dong Jin, Jing Yang, Shu-sheng Wu, Jianguo Xu

**Affiliations:** 1 State Key Laboratory of Infectious Disease Prevention and Control, National Institute for Communicable Disease Control and Prevention, Collaborative Innovation Center for Diagnosis and Treatment of Infectious Diseases, Chinese Center for Disease Control and Prevention, 102206 Changping, Beijing China; 2 Shanghai Institute for Emerging and Re-emerging infectious diseases, Shanghai Public Health Clinical Center, 201508 Shanghai, China; 3 Yushu Prefecture Center for Disease Control and Prevention, 815000 Yushu, Qinghai province China

## Abstract

Wildlife has been considered the main source of novel viruses causing emerging infectious diseases. *Marmota himalayana* is endemic to the Qinghai–Tibetan Plateau, China. Here, based on a high-throughput method using Illumina RNA sequencing, we studied the RNA virome of *M. himalayana* and discovered multiple novel viruses, especially picobirnaviruses (PBVs), which have a bi-segmented genome and belong to the family *Picobirnaviridae*. A total of 63% of the viral contigs corresponded to PBVs, comprising 274 segment 1 and 56 segment 2 sequences. Unexpectedly, four unsegmented PBV genomes were also detected and confirmed by PCR and resequencing. According to the phylogenetic analysis, the following nine PBV assortment types are proposed: C1:GI, C2:GIV, C4:GI, C4:GV, C5:GI, C7:GI, C8:GIV, C8:GV and C8:GII. We hypothesize a model of segmentation for the PBV genome, mediated by a 6-bp direct repeat sequence, GAAAGG. The model is supported by detection of the segmentation-associated sequence GAAAGG not only in the 5′ untranslated regions of segment 1 (221 in 289) and segment 2 (57 in 80) of bi-segmented PBVs but also in the 5′ untranslated regions and junction sequences between the capsid and RdRp genes of unsegmented PBVs. Therefore, with RNA sequencing, we found an unexpected biodiversity of PBVs in *M. himalayana*, indicating that *M. himalayana* is a special host for PBVs. We also proposed a putative model of how bi-segmented PBVs could be converted into unsegmented PBVs, which sheds new light on the processes of RNA virus genome evolution.

## Introduction

There have been frequent outbreaks of emerging infectious diseases caused by viruses of animal origin, such as the emergence of the Ebola virus in West Africa and the Middle East respiratory syndrome coronavirus in the Middle East^1, 2^. Investigations of the source of these novel viral infections have highlighted wildlife as a reservoir of zoonotic viruses^3, 4^. *Marmota himalayana* is a species endemic to the Qinghai–Tibetan Plateau that belongs to the family Sciuridae in the order Rodentia. It is considered a major reservoir of *Yersinia pestis*^5^. A novel hepatovirus and astrovirus have recently been detected in marmot^6, 7^.

Segmented RNA viruses are widespread in nature and include important human, animal and plant pathogens, such as influenza virus and tick-borne Jingmen virus. Viruses with segmented and unsegmented genomes typically belong to different viral families^8^. Unsegmented foot-and-mouth disease virus reportedly undergoes segmentation into two RNAs during prolonged cell culture^9^. Investigation of the recently discovered tick-borne Jingmen virus has revealed that a segmented RNA virus has a genome derived in part from unsegmented viral ancestors^10^. However, the mechanism by which an unsegmented RNA virus undergoes genome segmentation into a segmented virus is unclear.

Picobirnaviruses (PBVs) are small, non-enveloped, bi-segmented double-stranded RNA viruses of the family *Picobirnavirdae*^11, 12^. Segment 1 (2.2–2.7 kb) contains two open reading frames (ORF1 and ORF2) encoding a hydrophilic protein with unknown function and the capsid protein, respectively, whereas segment 2 (1.2–1.9 kb) has a single ORF and encodes the viral RNA-dependent RNA polymerase (RdRp)^13–15^. A recent study reported a fused RNA segment for PBV in horse^16^. Based on the amino acid sequence of the RdRp, PBVs are classified into five genogroups: GI, GII^17^, GIII^18^, GIV and GV^16^. The intra-genogroup and inter-genogroup sequence similarities of the RdRp nucleic acid fragment range from 49 to 100% and 28 to 37%, respectively^17^. PBVs are frequently detected in fecal samples from mammals, birds, and wild animals, as well as environmental samples and in immunocompromised patients as an opportunistic diarrhea-causing pathogen^19, 20^. In this study, using RNA sequencing (RNA-seq), we studied the RNA virome of *M. himalayana* and identified and characterized novel PBVs in this species, including bi-segmented and unsegmented genomes of PBVs. A putative model of PBV genome segmentation was also proposed.

## Materials and methods

### Marmot sampling

**Fig. 1:**
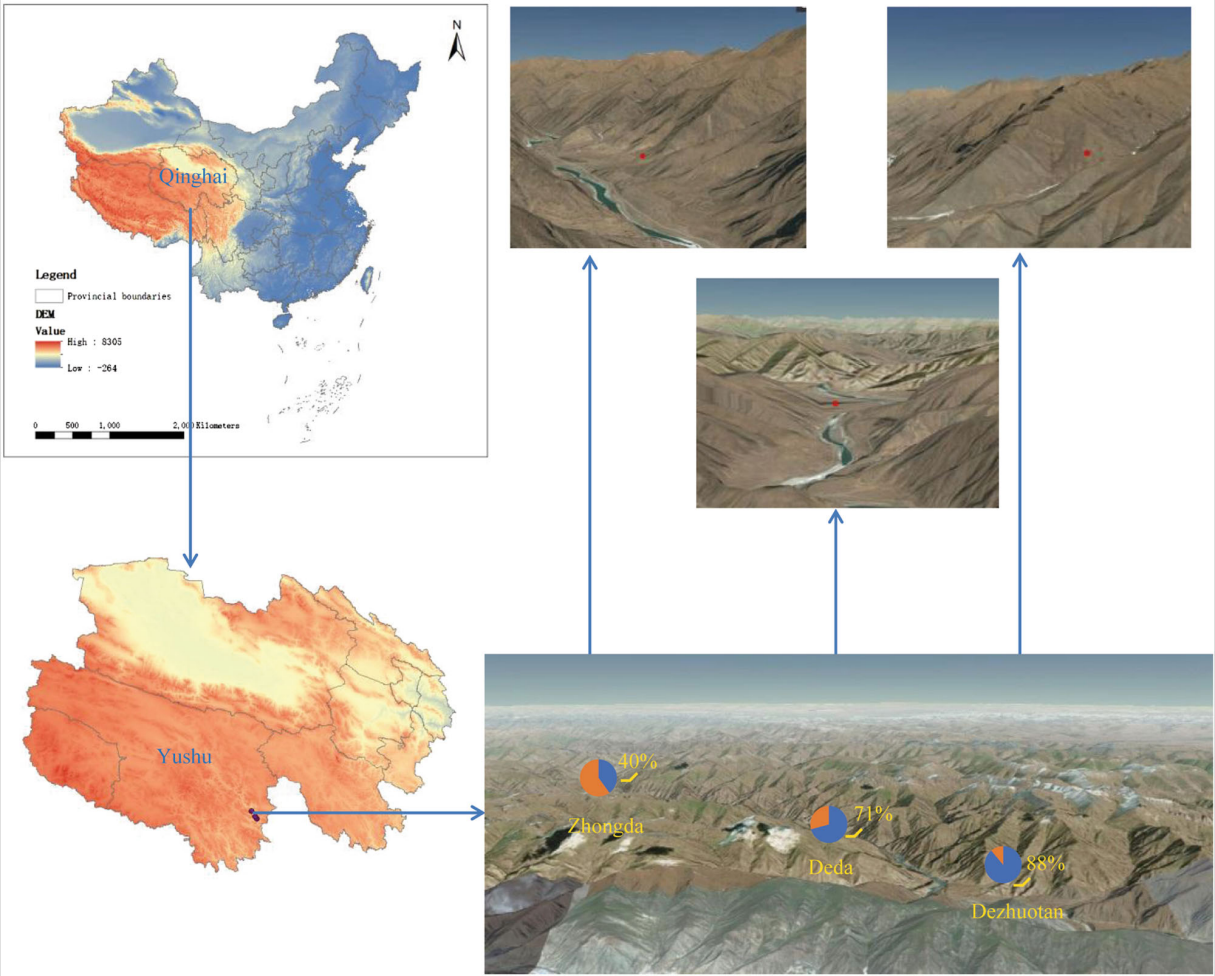
Map showing the location of the collection sites in Yushu, Qinghai province, China. Red dots are the collection sites. Positive rates in three locations are indicated

The 191* M. himalayana* were sampled from 29 June to 8 August 2013 as part of the animal plague surveillance program conducted in Yushu Tibetan autonomous prefecture, Qinghai province, China. The locations of the sampling were the counties of Zhongda (with an altitude of 3599.6 m above sea level), Dezhuotan (3025 m above sea level) and Deda (3625.6 m above sea level) in Yushu Tibetan autonomous prefecture, Qinghai province, China (Fig. 1). The marmots were captured live in cages in the field and sampled in the laboratory of the County’s Centre for Disease Control and Prevention. The sampling was performed in accordance with the protocol for the national plague surveillance program in animals. The intestinal contents were collected in 2 ml sterile tubes, which were kept at −80 °C until processing.

### Nucleic acid extraction

Each fecal sample was re-suspended (1:10, wt/vol) in PBS buffer and vortexed thoroughly. The suspension was clarified by centrifugation at 15,000×*g* for 10 min. The total viral nucleic acids were extracted with a QIAamp viral RNA mini kit (Qiagen, China) according to the manufacturer’s protocol. The concentration and quality of final RNA were examined using an ND-1000 UV spectrophotometer. These RNA were randomly merged into four pools for RNA-seq library construction and sequencing.

### Illumina sequencing and data analysis

The library preparation and sequencing steps were performed by BGI Tech (Shenzhen, China). Briefly, the total RNA was subjected to an rRNA removal step using a Ribo-Zero Magnetic Gold Kit (Epicentre, Madison, WI). The remaining RNA was then fragmented, reverse-transcribed, ends repaired, dA-tailed, adapter ligated, purified, and quantified with an Agilent 2100 Bioanalyzer and ABI StepOnePlus Real-Time PCR System. Pair-end (approximately 100 bp) sequencing of the RNA library was performed on the HiSeq 2000 platform (Illumina, San Diego, CA). Raw reads were quality trimmed and assembled *de novo* into contigs using the Trinity program^21^. The assembled contigs (>300 bp) were compared with the viral reference database and the GenBank non-redundant protein database using BLASTx search with an *E*-value of 10^−5^.

### Quantification of relative transcript abundances

The relative abundance of each transcript is presented as transcripts per million and corrects for the total number of reads as well as for the transcript length^22^. According to the procedure described, we first removed the rRNA reads from the data sets and then used the resulting rRNA contigs as a template for mapping using BOWTIE2^23^. Finally, the remaining reads from each library were then mapped onto the assembled transcripts and analyzed with RSEM^24^.

### Sequence confirmation and complete genome sequencing

For the confirmation of high-throughput sequencing results, we used nested RT-PCR to examine each potential viral sequence by specific primers. To obtain longer sequences or the complete genome, we used genome walking (Takara, Japan) and 5′ and 3′ rapid amplification of cDNA ends (Roche, USA) according to the manufacturer’s protocol. All specific primers used are available on request.

### Phylogenetic analysis

The viral amino acid sequences were predicted using the BioEdit program. Fifteen complete segment 1 sequences and 24 complete segment 2 sequences were downloaded from GenBank and used as reference sequences. Sequence alignment was performed using MAFFT version 7 with the E-INS-I algorithm. Phylogenetic trees were inferred using the maximum likelihood method implemented in PhyML version 3.0 with the WAG+Γ amino acid substitution model and a Subtree Pruning and Regrafting topology searching algorithm^25^.

### Nucleotide sequence accession numbers

Novel mammalian virus genome sequences in the study are at GenBank accession no. KY855432-KY855444. The RdRp and capsid sequences used in the study are at GenBank accession no. KY928683-KY928738 and KY928739-KY929012, respectively.

## Results

### Intestinal virome of *M. himalayana*

**Fig. 2:**
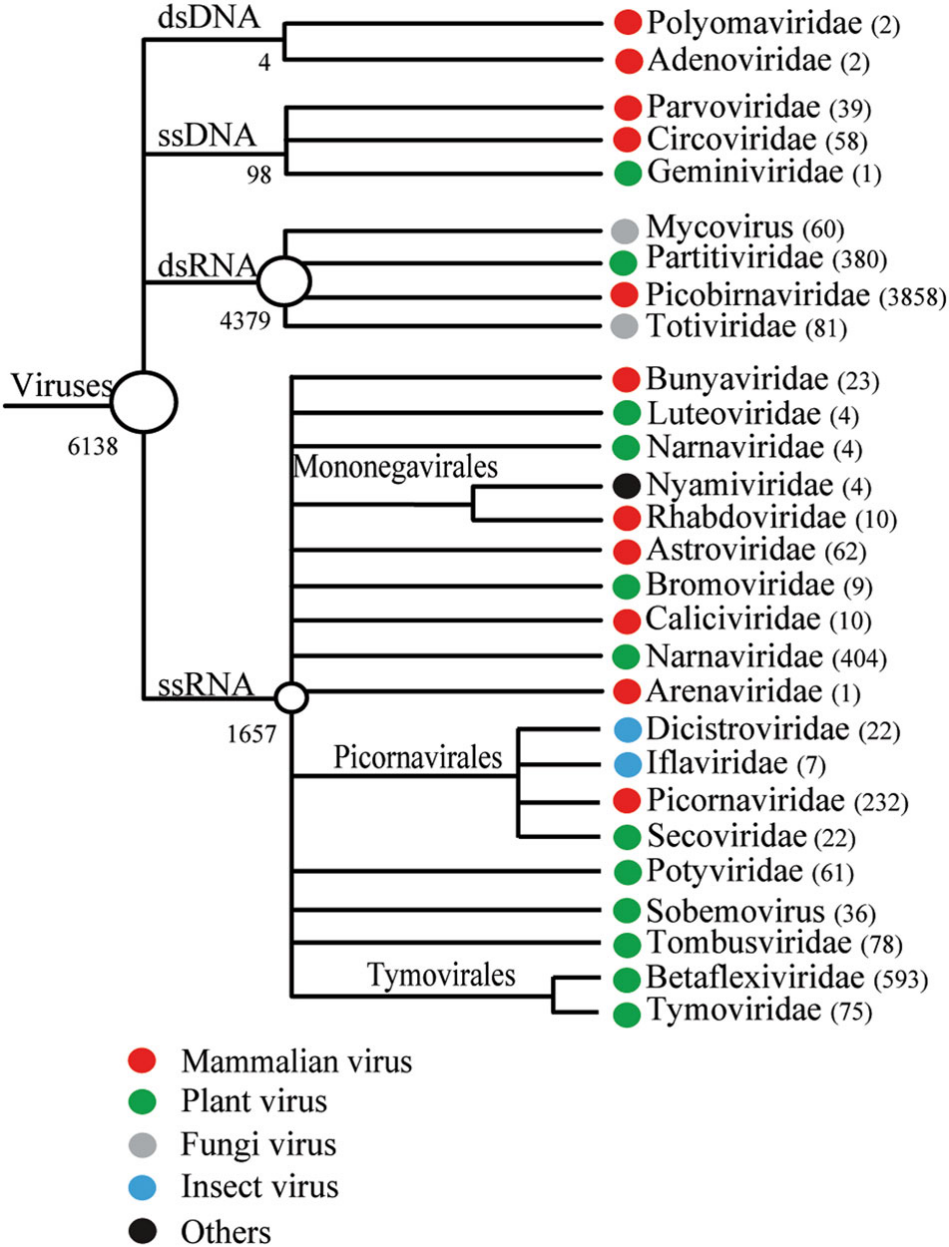
Virus families detected in the intestine of *Marmota himalayana*. Numbers of virus contigs are shown in parentheses

The intestinal contents of 191* M. himalayana* individuals were pooled and sequenced using Illumina HiSeq 2000, which generated a total of ~120 Gb of 100-bp end reads. The 379,618 high-quality >300-bp contigs obtained were subjected to BLASTx searches against a viral reference database and the GenBank non-redundant protein database using an *E*-value of 10^−5^. A total of 6138 contigs matched the sequences of viruses, classified to 28 virus families. Among them, 11 were mammalian-related virus families, including Picobirnavirdae (*n* = 3858), Picornaviridae (*n* = 232), Astroviridae (*n* = 62), Bunyaviridae (*n* = 23), Caliciviridae (*n* = 10), Mononegavirales (*n* = 14), and Arenaviridae (*n* = 1). The DNA virus families included Circoviridae (*n* = 58), Parvoviridae (*n* = 39), Adenoviridae (*n* = 2) and Polyomaviridae (*n* = 2) (Fig. 2). In addition, we obtained complete or nearly complete genome sequences for 13 new mammalian RNA viruses of three families, Picornaviridae, Astroviridae, and Caliciviridae (Supplementary Table S1). The polyproteins of Marmot sapalovirus HT5 and HT6 showed 54 and 56% amino acid sequence similarities to the genera *Enterovirus* and *Sapelovirus*, respectively, which are proposed new species of the *Enterovirus/Sapelovirus* supergroup (www.picornaviridae.com). The polyproteins of Marmot cardiovirus HT7 and Marmot mosavirus HT8 showed 59 and 46% amino acid sequence similarities to those of the viruses of the genera *Cardiovirus* and *Mosavirus*, respectively, which are considered new members of Cardiovirus and Mosavirus.

### Detection of unsegmented PBVs

**Fig. 3:**
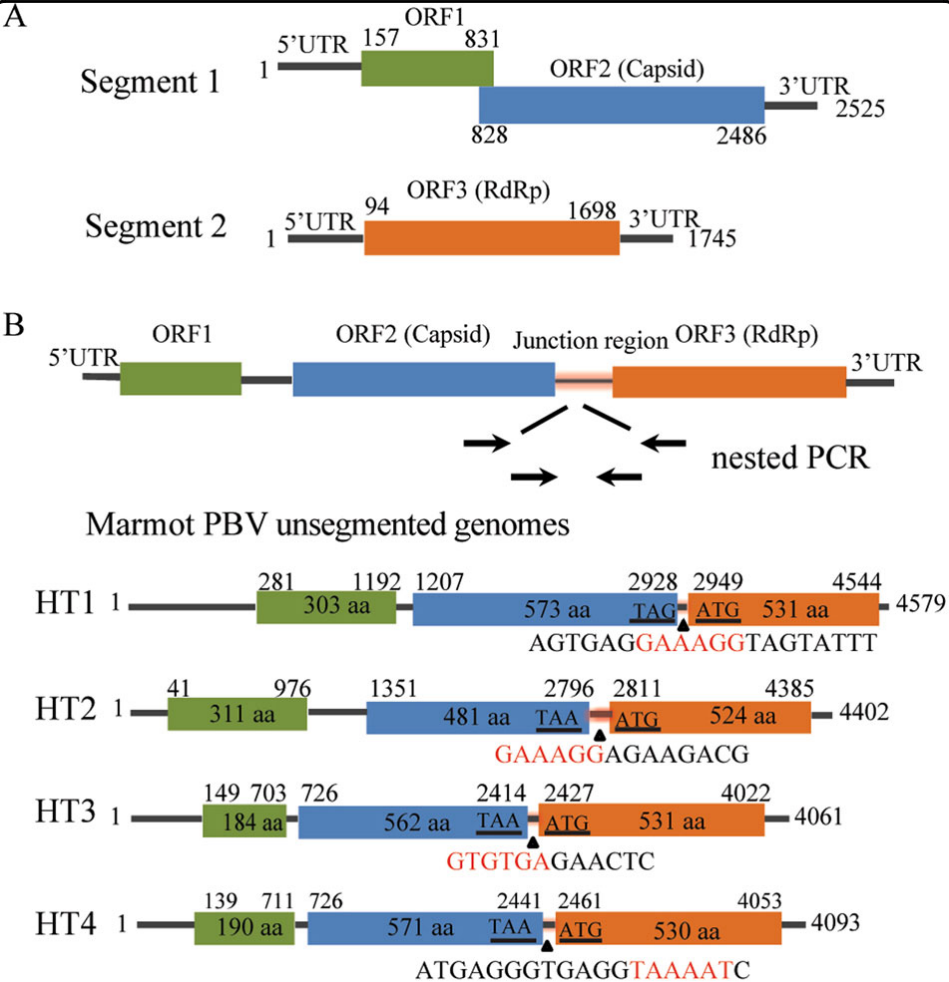
Genome organization of picobirnaviruses. **(a)** Schematic representation of the two segments of human picobirnavirus strain Hy005102 (GenBank accession no. NC_007026 and NC_007027). (B) The unsegmented genomes of picobirnaviruses detected in marmot. Arrows indicate the position and direction of primers for nested PCR. Stop codon of the capsid gene and the initiation codon of the RdRp are underlined. Junction sequences between segments 1 and 2 of bi-segmented picobirnaviruses are shown below the triangles. Segmentation-associated motifs are shown in red

BLASTx analysis showed that of 6138 virus contigs 3858 (62.9%) belonged to PBVs and exhibited 25–74% amino acid similarities. A total of 274 segment 1 and 56 segment 2 sequences were obtained. Unlike the conventional two-segment genomes of PBVs (Fig. 3a), we found four unsegmented genomes (named Marmot PBV HT1-4) (Fig. 3b, GenBank accession no. KY855428-KY855431). A nested polymerase chain reaction (PCR) bridging segment 1 and 2 was performed with two pairs of primers (Supplementary Table S2). The expected PCR product was sequenced, and the truth of the junction between both segments was confirmed. The four unsegmented genomes were also confirmed by re-sequencing. The junction sequences linking both segments of Marmot PBV HT1-4 were of 20, 14, 12, and 19-bp, respectively (Fig. 3b). Overall, 20 of 50 (40%), 17 of 24 (71%) and 104 of 117 (88%) intestinal samples from Zhongda, Deda, Dezhuotan, respectively, were positive for unsegmented PBVs (Fig. 1).

### Genome characterization of unsegmented PBVs

These unsegmented PBV sequences ranged from 4061 to 4579 bases in length with overall G + C contents of 39.9–45.4%. The 5′ untranslated regions (UTRs) (40–280 bases) were AU-rich (G + C contents of 28.2–32.6%). The 3′ UTRs (17–40 bases) contained G + C contents ranging from 37.1 to 64.7%. The genome of the unsegmented PBVs contained three ORFs, ORF1, ORF2 and ORF3 (Fig. 3b). ORF1 encoded a protein of 184–311 amino acids with unknown function. These proteins contained a different number of repetitions of the ExxRxNxxxE motif which was also observed in the corresponding protein in other known PBVs^26^. There were six ExxRxNxxxE motifs in the ORF1-encoded protein of Marmot PBV HT1 but only two ExxRxNxxxE motifs in those of Marmot PBV HT2-4. ORF2 encoded the capsid protein of 481–573 amino acids. These capsid proteins shared low (23–29%) amino acid identities with those of other PBV strains, being most closely related to PBV Equ2 (GenBank accession no. AKN50623). ORF3 encoded the RdRp of 524–531 amino acids. These RdRp shared 52–68% amino acid identities with those of other PBV strains. The amino acid similarities of the capsid and RdRp proteins of Marmot PBV HT1-4 were 53 and 69%, respectively. The pairwise amino acid identities of the capsid and RdRp regions of Marmot PBV HT1-4 suggested a high level of diversity among the unsegmented PBVs (Supplementary Table S3).

A recent study had reported one fused PBV genome Equ4 (GenBank accession no. KR902502) in diseased horse feces. An alignment result revealed that the highest amino acid similarity of capsid and RdRp between Marmot PBV HT1-4 and PBV Equ4 was 14 and 39%, respectively. There were four conserved motifs in RdRp regions among unsegmented PBVs, such as MFP, HGM(L)G(A)SGS, GDD and RALG at 242, 366, 405 and 472 sits, respectively, whereas no conserved motifs were found among the capsid proteins. These results suggested that unsegmented PBVs exhibited a high diversity.

### PBV assortment types

**Fig. 4:**
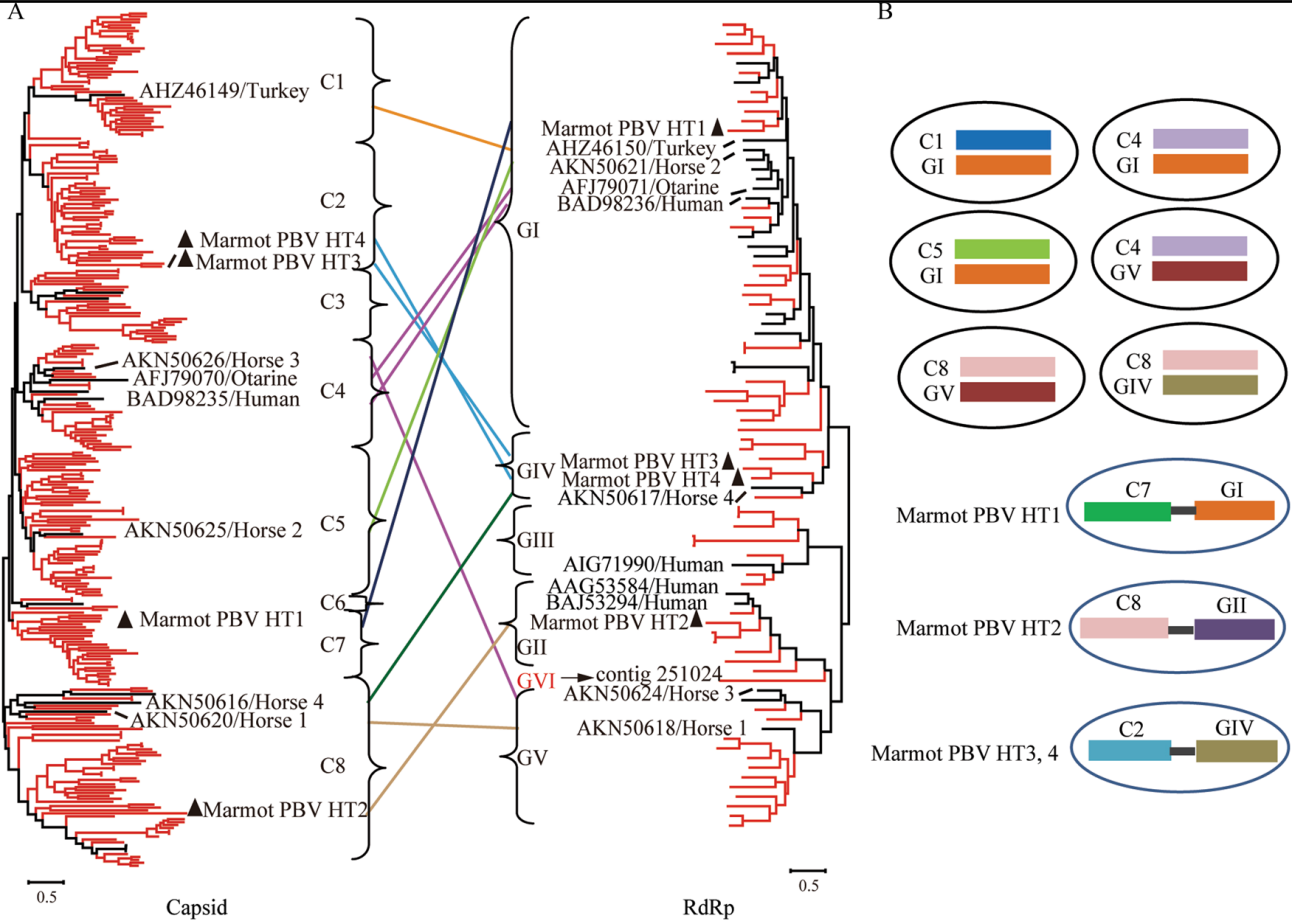
Maximum-likelihood phylogenetic trees and picobirnavirus assortment types. **a** The phylogenetic trees of the full-sequence capsid (left) and RdRp (right) proteins. Sequences of picobirnaviruses obtained from marmot are shown in red. Sequences of picobirnaviruses reported previously are shown in black. Unsegmented picobirnaviruses from marmot are indicated by black triangles. Capsid and RdRp sequences of picobirnaviruses that underwent assortment are linked by lines. **b** Proposed assortment types of bi-segmented and unsegmented picobirnaviruses

PBVs are classified into genogroups GI–GV. All five genogroups of RdRp were detected in marmot. In addition, an RdRp contig showed only 37% amino acid sequence similarity to those of the other genogroups. Thus, a new genogroup of RdRp named GVI was identified (Fig. 4a). The intra members of all six genogroups of RdRp shared similarity >42.41% at the amino acid level. The intergroup amino acid similarity for all RdRp groups ranged from 25.49–40.86% (Supplementary Figure S1). The RdRps of unsegmented Marmot PBV HT1-4 were grouped into GI, GII, and GIV, respectively (Fig. 4a).

By phylogenetic analysis of 293 complete capsid sequences from marmot and other hosts, eight clusters of capsids (C1-8) were proposed (Fig. 4a). The capsid sequences of unsegmented Marmot PBV HT1-4 were classified into clusters C2, C7, and C8; Marmot PBV HT3 and four were in C2 (Fig. 4a).

Based on the genome sequences of isolated PBVs and the unsegmented genomes of Marmot PBVs^14, 15, 27–29^, we propose nine PBV assortment types (Fig. 4b): C1:GI, C2:GIV, C4:GI, C4:GV, C5:GI, C7:GI, C8:GIV, C8:GV, and C8:GII. Clearly, the genogroup G1 for RdRp could assort with clusters C1, C4, C5, and C7 for capsids (Fig. 4b). Cluster C8 for capsids could assort with the RdRp of genogroups GIV, GV, and GII (Fig. 4b). Unsegmented Marmot PBVs exhibited three assortment types—HT1 for C7:GI, HT2 for C8:GII, and HT3 and HT4 for C2:GIV (Fig. 4b); this was not observed in bi-segmented PBVs.

### Direct repeat sequence segmentation model

Because both unsegmented and bi-segmented PBVs were identified in *M. himalayana*, we hypothesized that unsegmented PBVs underwent genome segmentation into segmented PBVs. First, the junction sequences linking the capsid and RdRp regions of unsegmented Marmot PBV HT1-4 were analyzed for possible segmentation-associated motifs. An alignment of these junction sequences and the 5′ UTR of the segment 2 showed three conserved motifs: TAAAAT, GAAAGG, and GTGTGA. Marmot PBV HT1 and HT2 have GAAAGG at the junction between the capsid and RdRp genes (Fig. 3b), whereas Marmot PBV HT3 and HT4 have GTGTGA or TAAAAT (Fig. 3b). These conserved motifs were also identified in 63 segment 2 of segmented PBVs in *M. himalayana* and other hosts (Supplementary Figure S4). However, an alignment of the junction sequences of unsegmented PBVs and the 3′ UTR of the segment 1 detected no conserved motifs. These findings indicated that the three conserved motifs might be involved in the segmentation of PBVs.

**Fig. 5:**
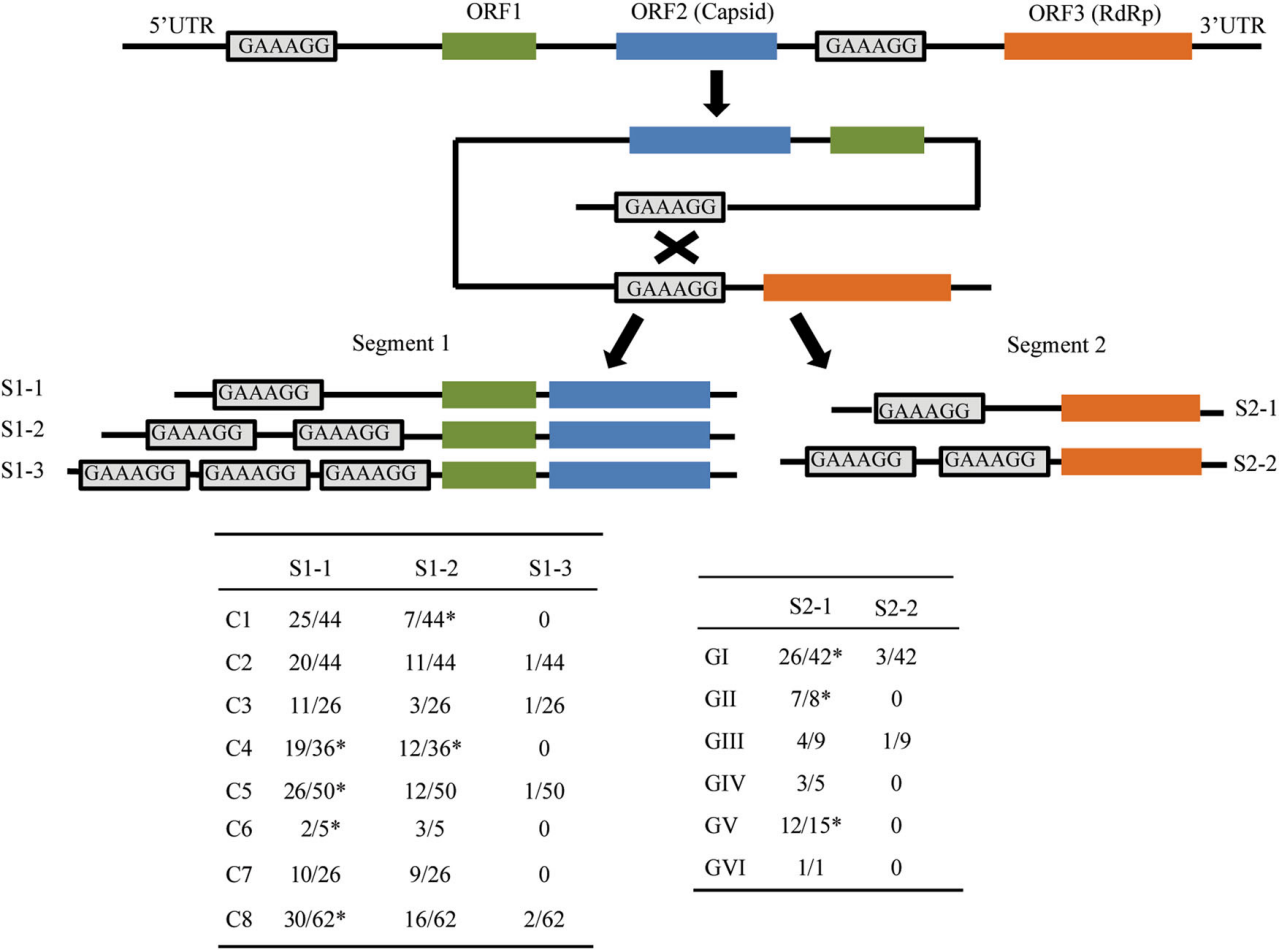
Model of segmentation of unsegmented picobirnaviruses. Relative positions of the 6-bp direct repeat sequence GAAAGG in the 5′ UTR and junction between the capsid and RdRp genes of unsegmented Marmot picobirnaviruses are indicated by rectangles. A schematic of the three types of segment 1 harboring GAAAGG (S1-1-3) and two types of segment 2 harboring GAAAGG (S2-1-2) from bi-segmented picobirnaviruses. The proportions of S1-1-3 and S2-1-2 among the total sequences analyzed, together with their genotypes, are shown in the following tables. *The published segment 1 sequences (GenBank accession no. KJ495689; KF861768; JQ776551; AB186897; KR902506; KC692367; KR902502; KR902504; KF861770; KF861771; KF861772). The published segment 2 sequences (GenBank accession no. JQ776552; AAG53583; AHX00960; ACT64131; AFK81927; BAJ53294; AHZ46150; KR902507; KR902503)

We next analyzed the UTR of the PBV sequences obtained in this study and those reported previously. Strikingly, most segment 1 and 2 sequences had the GAAAGG in 5′ UTRs. In particular, 57 segment 2 sequences (distributed among all six genotypes) had GAAAGG in the 5′ UTR (Fig. 5). Of 289 segment 1 sequences analyzed, 221 (distributed among all eight clusters) had the GAAAGG motif in the 5′ UTR (Fig. 5). Notably, 78 segment 1 sequences had two or three copies of GAAAGG in the 5′ UTR, and four segment 2 sequences had two copies of GAAAGG in their 5′ UTRs (Fig. 5). Furthermore, Marmot PBV HT1 and HT2 had GAAAGG in their 5′ UTRs. Therefore, we propose a 6-bp direct repeat sequence GAAAGG-mediated segmentation model for Marmot unsegmented PBVs (Fig. 5).

We also detected GTGTGA or TAAAAT in the 5′ UTR of segment 2 (Supplementary Figure S2). Ten segment 2 sequences had TAAAAT in their 5′ UTR while five had both the TAAAAT and the GAAAGG in their 5′ UTR. Three segment 2 sequences have GTGTGA in their 5′ UTRs. Neither the GTGTGA nor the TAAAAT motif was detected in the 5′ UTR of segment 1 or unsegmented PBVs from marmot.

## Discussion

RNA-seq has been used to study the transcriptome in various cells and tissues based on high-throughput sequencing. Recently, this technique has been successfully used to discover a large number of novel RNA viruses^30, 31^. In the present study, we used RNA-seq to study RNA virome in *M. himalayana* fecal samples and identified novel viruses belonging to different families, such as *Picobirnaviridae*, *Picornaviridae*, *Astroviridae*, and *Caliciviridae* (Fig. 2 and Supplementary Table S1).

PBV sequences predominated in the intestine of *M. himalayana*. In addition, we also detected four unsegmented PBV genomes (named Marmot PBV HT1-4). Therefore, *M. himalayana* should be considered a special animal reservoir of PBVs. We failed to isolate unsegmented PBVs from the fecal samples of *M. himalayana*, hampering further biological research on unsegmented PBVs.

All of the PBV sequences obtained from *M. himalayana*, together with published reference PBV sequences, were divided into six genogroups for the RdRp genes and eight clusters for the capsid genes. A phylogenetic analysis (Fig. 4a) suggested recombination between the capsid and RdRp genes. This recombination was reflected not only in the different phylogenetic trees between the two genes but also in the marked diversity of capsid protein sequences. The four Marmot PBVs were dispersed within the segmented PBVs in phylogenetic trees (Fig. 4a), which suggests that multiple segmentation events might have occurred frequently.

The amino acid alignment analysis of RdRp sequences obtained in marmot with other RdRp sequences of PBVs showed three patterns: GI/GIV, GIII/GV/GVI, and GIII. The intra sequences of GI/GIV had higher conservation compared with that of GIII/GV/GVI, and GIII (Supplementary Figure S3). A motif GDD at the 472 site was conserved among all RdRp regions, whereas no conserved motifs were found among the capsid proteins. Of interest, the number of ExxRxNxxxE motifs was variable in ORF1-encoded proteins of marmot PBV sequences, ranging from 0 to 16. Half (~56%) of these proteins had three to six ExxRxNxxxE motifs, whereas ten of them did not possess this motif (Supplementary Figure S4).

Screening for1 segmentation-associated motifs revealed GAAAGG in the 5′ UTR and junction region between the capsid and RdRp genes of the unsegmented Marmot PBV HT1 and HT2 (Fig. 5). Therefore, we proposed that the segmentation of unsegmented PBVs was mediated by GAAAGG (Fig. 5). This is supported by the finding that 57 of 80 segment 2 sequences (covering all six genotypes of RdRp) had the GAAAGG motif in their 5′ UTRs (Fig. 5). This motif was also detected in 221 of 289 segment 1 sequences (covering all eight clusters classified) (Fig. 5). Direct repeat sequences have been identified in DNA viruses, RNA viruses or phages^10, 32–37^. Direct repeat sequences evolved through multiple duplications, deletions, and mutations of a primordial sequence element in tick-borne flaviviruses^33^. Interestingly, of 289 segment 1 sequences with the GAAAGG motif, 143, 73, and 5 had one, two, and three copies, respectively, in their 5′ UTR (Fig. 5). Among the 80 segment 2 sequences with the GAAAGG motif, 55 and 4 had one and two copies, respectively, in their 5′ UTR (Fig. 5). The specific mechanism by which GAAAGG-mediated genome segmentation occurs needs to be further studied.

In conclusion, we have identified a number of previously unknown RNA viruses in the intestine of *M. himalayana* on the Qinghai–Tibetan Plateau, China, especially novel PBVs. Discovering and characterizing both bi-segmented and unsegmented PBVs in *M. himalayana* encourages us to rethink the evolution and classification of PBVs. In addition, the putative model of PBV segmentation provides a new perspective on genome segmentation in RNA viruses.

## Electronic supplementary material

Supplementary Figure S1

Supplementary Figure S2

Supplementary Figure S3

Supplementary Figure S4

Supplementary Table S1

Supplementary Table S2

Supplementary Table S3
