# Supplementary material for: Marmota himalayana in the Qinghai–Tibetan plateau as a special host for bi-segmented and unsegmented picobirnaviruses
Source: Emerg Microbes Infect. 2018 Mar 7;7:20. doi: 10.1038/s41426-018-0020-6 (PMC5841229; doi:10.1038/s41426-018-0020-6)

**Supplementary Figure S1 Pairwise inter- and intra-genogroup amino acid sequences similarity of RdRp proteins of picobirnaviruses. (A)** the intra-genogroup amino acid sequences similarity of RdRp. **(B)** the inter-genogroup amino acid sequences similarity of RdRp.


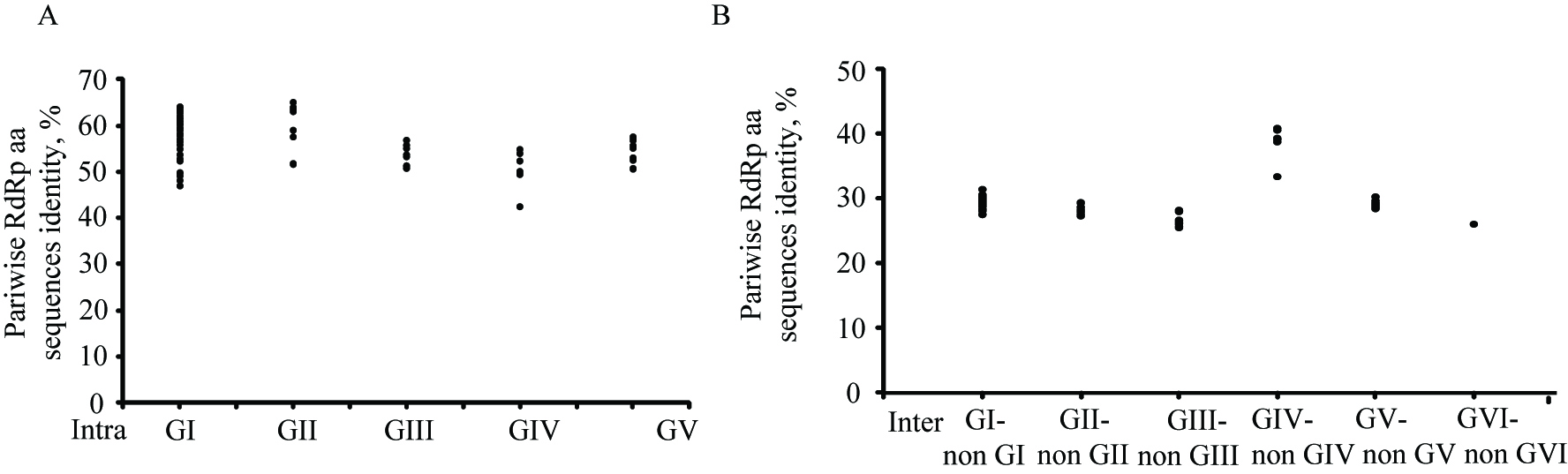

Supplement: Supplementary file 1 — Supplementary Figure S1 [file 41426_2018_20_MOESM1_ESM.docx]
