# Supplementary material for: Marmota himalayana in the Qinghai–Tibetan plateau as a special host for bi-segmented and unsegmented picobirnaviruses
Source: Emerg Microbes Infect. 2018 Mar 7;7:20. doi: 10.1038/s41426-018-0020-6 (PMC5841229; doi:10.1038/s41426-018-0020-6)

**Supplementary Figure 2S Maximum-likelihood phylogenetic tree of RdRp proteins in segment 2 sequences with 6-bp direct repeat sequence.** The relative position of three types direct repeat sequence in the 5’ UTR of RdRp are marked with colored circle, invented triangle, and square. Their subtypes of 6-bp direct repeat sequence with a single nucleotide change are labeled with number. The RdRp sequences from Marmot unsegmented picobirnaviruses are in red. The picobirnaviruses isolated from other hosts are also included, such as porcine (AHZ59999), otarine (AFJ79071), turkey (AHZ46150), monkey (AFK81927), bovine (ACT64131), human (AHX00960, BAD98236, AAG53583, AIG71990, AAG53584, BAJ53294), horse (AKN50618, AKN50621, AKN50624, KR902502).


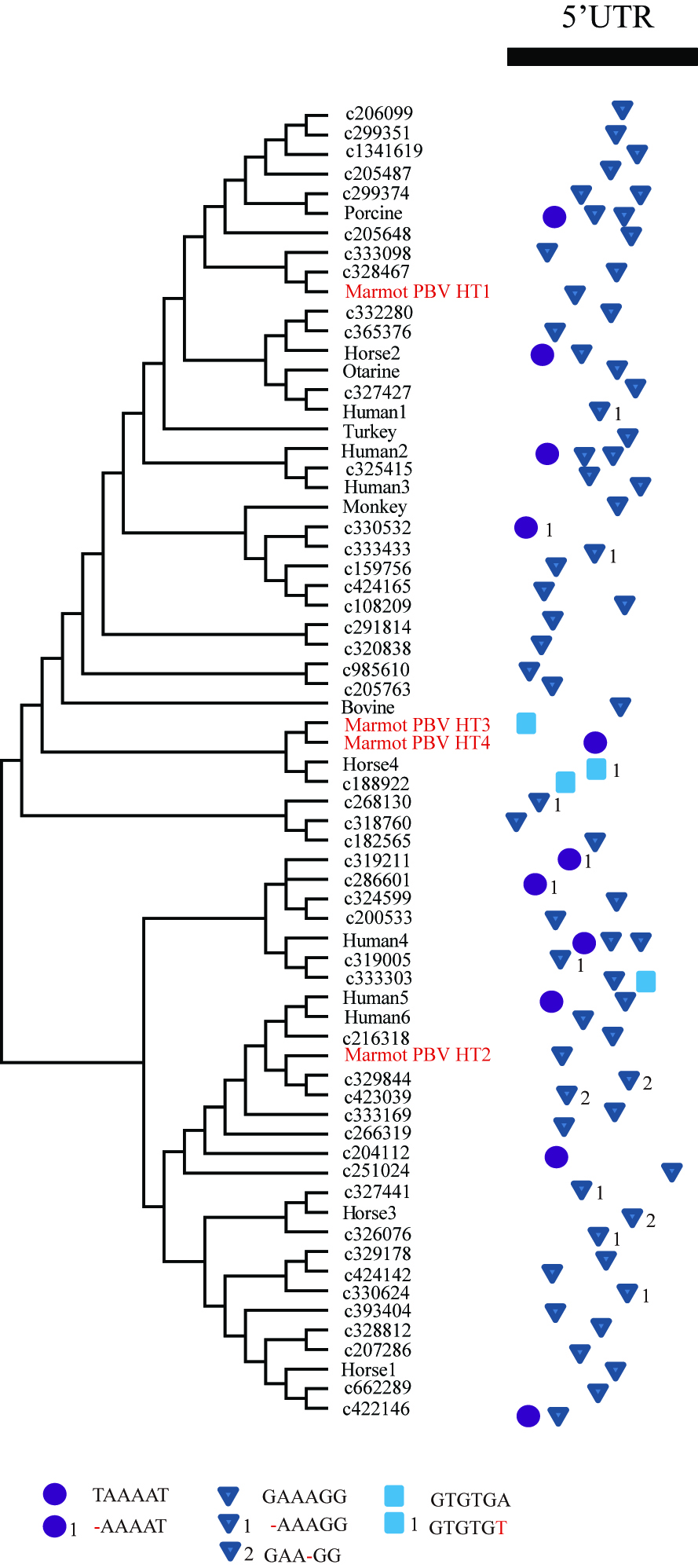

Supplement: Supplementary file 2 — Supplementary Figure S2 [file 41426_2018_20_MOESM2_ESM.docx]
