# Supplementary material for: Marmota himalayana in the Qinghai–Tibetan plateau as a special host for bi-segmented and unsegmented picobirnaviruses
Source: Emerg Microbes Infect. 2018 Mar 7;7:20. doi: 10.1038/s41426-018-0020-6 (PMC5841229; doi:10.1038/s41426-018-0020-6)

**Supplementary Figure S3 Amino acid alignment analysis of RdRp sequences obtained in marmot with other RdRp sequences of picobirnaviruses.** It shows the relative position of insertion and deletion. The conserved regions are indicated by shading and the remarkable amino acid conserved sits are marked by different orange according to the degree of conservation. The reference sequence of RdRp for alignment is from human (BAD98236).


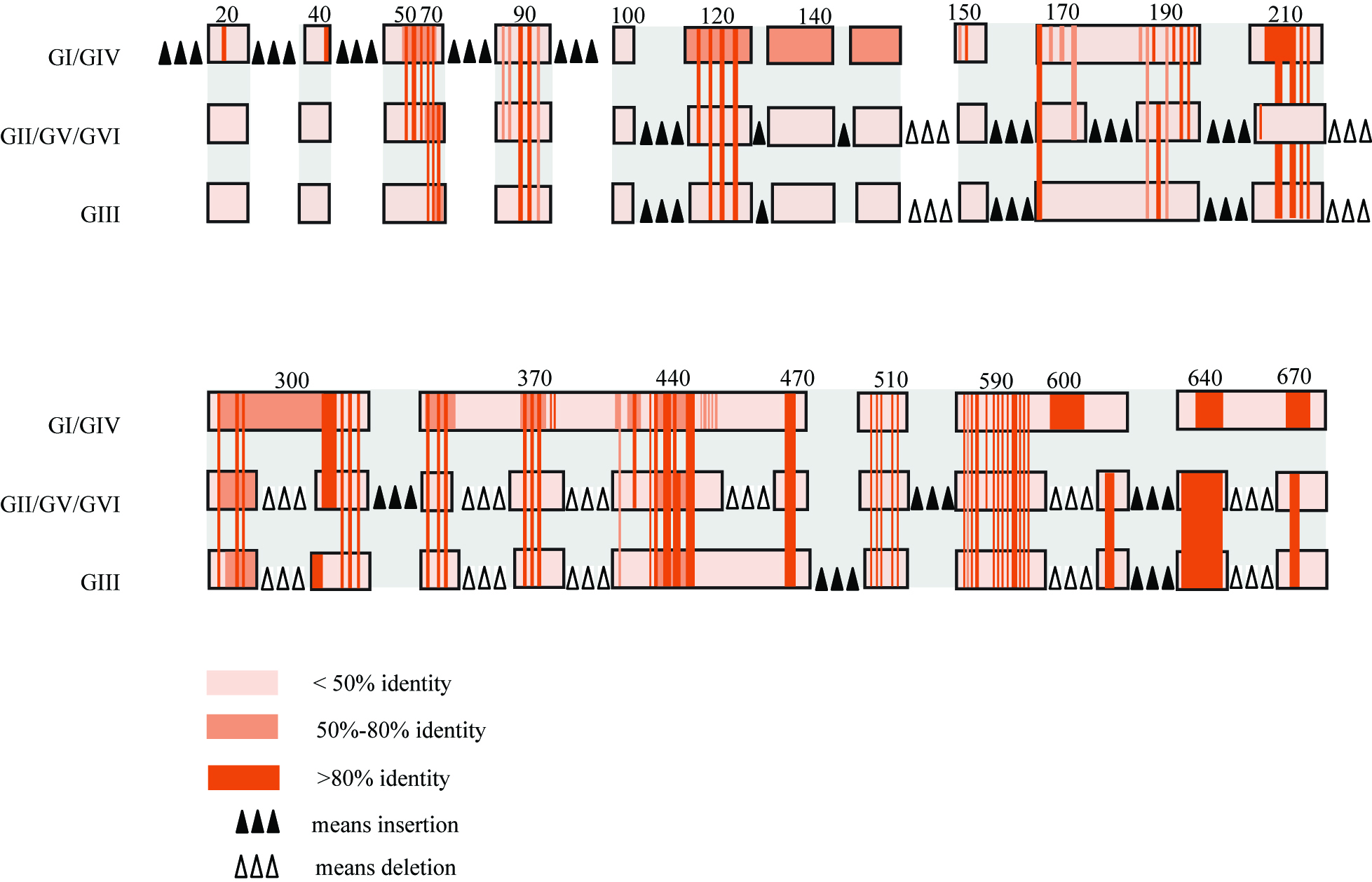

Supplement: Supplementary file 3 — Supplementary Figure S3 [file 41426_2018_20_MOESM3_ESM.docx]
