# Supplementary material for: Marmota himalayana in the Qinghai–Tibetan plateau as a special host for bi-segmented and unsegmented picobirnaviruses
Source: Emerg Microbes Infect. 2018 Mar 7;7:20. doi: 10.1038/s41426-018-0020-6 (PMC5841229; doi:10.1038/s41426-018-0020-6)

**Supplementary Figure S4** **The numbers of the motif ExxRxNxxxE (x=any amino acid) in ORF1-encoded proteins of marmot.**


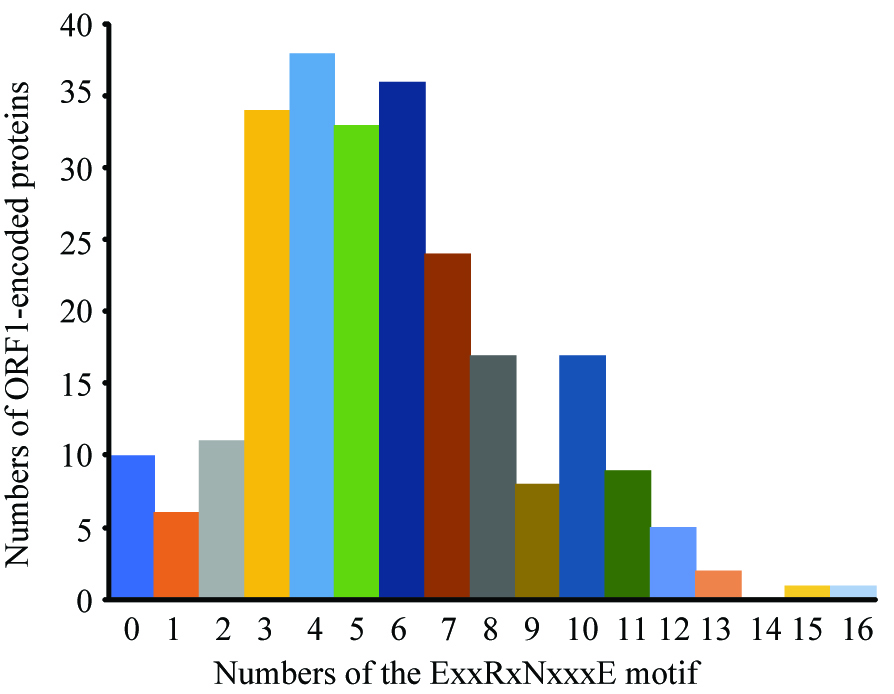

Supplement: Supplementary file 4 — Supplementary Figure S4 [file 41426_2018_20_MOESM4_ESM.docx]
