# Supplementary material for: Marmota himalayana in the Qinghai–Tibetan plateau as a special host for bi-segmented and unsegmented picobirnaviruses
Source: Emerg Microbes Infect. 2018 Mar 7;7:20. doi: 10.1038/s41426-018-0020-6 (PMC5841229; doi:10.1038/s41426-018-0020-6)
Supplement: Supplementary file 5 — Supplementary Table S1 [file 41426_2018_20_MOESM5_ESM.docx]

**Supplementary Table S1 List of the novel mammalian viruses discovered in *Marmota himalayana*.**

Name Genbank Genome Classification Abundance

No. length(bp) (Family/genus) (TPM)

| Marmot sapelovirus HT5 | KY855432 | 7726 | Picornaviridae/sapelovirus | 46.32 |
| --- | --- | --- | --- | --- |
| Marmot sapelovirus HT6 | KY855433 | 7834 | Picornaviridae/sapelovirus | 782.73 |
| Marmot cardiovirus HT7 | KY855434 | 6253* | Picornaviridae/cardiovirus | 57.62 |
| Marmot mosavirus HT8 | KY855435 | 8170 | Picornaviridae/mosavirus | 20.89 |
| Marmot kobuvirus HT9 | KY855436 | 8283 | Picornavirus/kobuvirus | 2.02 |
| Marmot astrovirus HT10 | KY855437 | 6739 | Astroviridae/mamastrovirus | 1936.32 |
| Marmot astrovirus HT11 | KY855438 | 6708 | Astroviridae/mamastrovirus | 7737.66 |
| Marmot astrovirus HT12 | KY855439 | 6466 | Astroviridae/mamastrovirus | 389.577 |
| Marmot astrovirus HT13 | KY855440 | 6420 | Astroviridae/mamastrovirus | 4286.64 |
| Marmot astrovirus HT14 | KY855441 | 6420 | Astroviridae/mamastrovirus | 528.89 |
| Marmot astrovirus HT15 | KY855442 | 6420 | Astroviridae/mamastrovirus | 3098.63 |
| Marmot norovirus HT16 | KY855443 | 6453 | Caliciviridae/norovirus | 36.9 |
| Marmot sapovirus HT17 | KY855444 | 5915* | Caliciviridae/sapovirus | 11.12 |

* Partial sequences
