# Supplementary material for: Marmota himalayana in the Qinghai–Tibetan plateau as a special host for bi-segmented and unsegmented picobirnaviruses
Source: Emerg Microbes Infect. 2018 Mar 7;7:20. doi: 10.1038/s41426-018-0020-6 (PMC5841229; doi:10.1038/s41426-018-0020-6)
Supplement: Supplementary file 6 — Supplementary Table S2 [file 41426_2018_20_MOESM6_ESM.docx]

**Supplementary Table S2 The primers used for verification the junction between the RdRp and capsid region.**

Primer name sequence (5’-3’)

| mpbv1-F1 | CCGATGCTGAATGATAAG |
| --- | --- |
| mpbv1-R1 | GATCCCAATGCCTCTGACCAC |
| mpbv1-F2 | GACTACGGTACAACAGTAC |
| mpbv1-R2 | CTTTGCTTTGCATATCAGTTTC |
| mpbv2-F1 | GGTATACACTGCTTCAGTCCCTC |
| mpbv2-R1 | ACTACCGAGGTATACGCAAGAGG |
| mpbv2-F2 | CGCGCACGCTCCGTCTTTAATAT |
| mpbv2-R2 | TCAGAGGAGGAATAGAGAGGTTC |
| mpbv3-F1 | CTTGTAACTGGTGTTCATGTATG |
| mpbv3-R1 | GGACGTTGAATTGATAATGGTCC |
| mpbv3-F2 | CTTCGCAAACTTCAAGTATGCTC |
| mpbv3-R2 | GGTCCTCACCTCACAGTCGTAAC |
| mpbv4-F1 | GGTGCAGACTGGCTGCCTTTGCT |
| mpbv4-R1 | ACCACCCACTAGTCTACGCACC |
| mpbv4-F2 | CCGAGGTTAAAATGTGGAGCTAT |
| mpbv4-R2 | TGACATTGGTCCTACTTTGGAAG |
