# Supplementary material for: Marmota himalayana in the Qinghai–Tibetan plateau as a special host for bi-segmented and unsegmented picobirnaviruses
Source: Emerg Microbes Infect. 2018 Mar 7;7:20. doi: 10.1038/s41426-018-0020-6 (PMC5841229; doi:10.1038/s41426-018-0020-6)
Supplement: Supplementary file 7 — Supplementary Table S3 [file 41426_2018_20_MOESM7_ESM.docx]

**Supplementary Table S3 Pairwise amino acid identities of the capsid (upper right) and RdRp (lower left) regions of Marmot picobirnaviruses.**

Marmot PBV

HT1 HT2 HT3 HT4

| Marmot PBV | HT1 |  | 0.246 | 0.266 | 0.224 |
| --- | --- | --- | --- | --- | --- |
|  | HT2 | 0.262 |  | 0.190 | 0.192 |
|  | HT3 | 0.520 | 0.271 |  | 0.599 |
|  | HT4 | 0.552 | 0.275 | 0.695 |  |
